# Phytase in the diets of broilers: technical-economic evaluation

**DOI:** 10.5713/ab.250612

**Published:** 2025-11-25

**Authors:** Danilo de Souza Sanches, Charles Kiefer, Ricardo Carneiro Brumatti, Karina Marcia Ribeiro de Souza Nascimento, Anderson Corassa, Luan Sousa dos Santos, Gislaine da Cunha de Andrade

**Affiliations:** 1 Veterinary and Animal Science Department, Federal University of Mato Grosso do Sul, Campo Grande, Brazil; 2 Agrarian and Environmental Sciences Institute, Federal University of Mato Grosso, Sinop, Brazil

**Keywords:** Exogenous Enzyme, Nutritional Cost, Performance, Poultry Farming

## Abstract

**Objective:**

The objective of this study was to evaluate the effects of phytase in broiler diets on zootechnical performance and nutritional cost through a technical economic analysis combined with a systematic review and meta-analysis.

**Methods:**

A database was created by collecting scientific articles published between 2018 and 2023 that investigated broilers supplemented with phytase. The PRISMA (Preferred Reporting Items for Systematic Reviews and Meta-Analyses) flowchart was used to select the articles. A total of 3,483 publications were identified, of which 17 were selected. Performance data from the articles and qualitative factors (broiler strain, diets, and phosphorus reduction) were collected, tabulated, and used in economic equations. Meta-analyses were conducted to assess effect size, heterogeneity, and publication bias for performance variables among the groups positive control (DB) vs. reduced P diet without phytase (DRP), DB vs. reduced P diet with phytase (DRP+PHY), and DRP vs. DRP+PHY.

**Results:**

Seventeen studies were selected, totaling 16,151 broilers evaluated, with average initial and final weights of 44.0±2.0 g and 2,731±0.6 g, respectively, receiving 1,664 FTU/kg of phytase and a 0.15% reduction in digestible phosphorus. Overall, the results indicate that phytase supplementation improves broiler performance (p<0.05), whereas DRP without phytase impair it (p<0.05). These findings were reinforced by our meta-analysis, which identified a significant effect size (p_SMD_<0.001) for all variables studied, high heterogeneity among studies (p<0.001, I^2^ = 96%), with broiler strain sending as the main source of variation. No publication bias was detected across the evaluated variables. The broilers subjected to DRP+PHY had a lower cost/head and greater final weight than those fed BD and DRP, respectively. In addition, DRP+PHY reduced the feed cost per ton of broilers by −1.56%.

**Conclusion:**

Reducing phosphorus in diets without phytase impairs broiler performance, while supplementing phytase in low-phosphorus diets improves performance and reduces nutritional costs, saving approximately US$9.52 per ton of broilers.

## INTRODUCTION

Approximately two-thirds of the total phosphorus (P) contained in the plant ingredients that compose the diets of birds is complexed with phytate [1]. For this P to be used by the bird, it is necessary that the phytate be hydrolyzed by phytase. Although there is slight activity of phytase in the intestinal mucosa of birds, this activity is insufficient to break the molecule, making P unavailable [2]. Thus, exogenous phytase has been used in diets to hydrolyze phytate in the gastrointestinal tract, making P and other nutrients available for absorption by birds [3].

The scientific literature shows that the inclusion of phytase in diets for broilers can improve the digestibility of P and calcium [4,5], amino acids [6,7], nitrogen and energy [8,9], reduce P excretion [5] and diet cost [10–12], without impairing zootechnical performance, bone structure and carcass and cut yield [2,13]. Several studies using different sources of phytases (bacterial and fungal) in the feeding of nonruminant animals have been reviewed in recent years by Walk et al [14], Abd el-hack et al [2], and Selle et al [15], who demonstrated the positive effects of the enzyme on productive, economic and environmental characteristics.

However, it is necessary to update its effects on contemporary production from the perspective that the basic ingredients that make up the diets have been suffering constant price fluctuations, increasing the costs of the diets. In addition, there is a lack of information in the literature regarding the technical-economic impact and the real financial returns that the inclusion of phytase can promote in the feeding of broilers.

A technique used to update the literature is meta-analysis, which quantitatively integrates data from different studies, increasing the robustness and applicability of the results. Unlike isolated experiments, which are limited by their specific conditions, meta-analytic methods systematically account for heterogeneity among studies, providing a broader understanding of the topic [16].

Unlike conventional meta-analyses, our study constructed the database by considering three treatments from each selected study (DB: positive control; DRP: reduced P diet without phytase; DRP+PHY: reduced P diet with phytase). Thus, the meta-analytical responses regarding the effect of phytase were expanded from different perspectives. In addition, new economic insights were obtained, since methodologies that simulate techno-economic evaluations using performance data obtained through systematic review and meta-analysis are pioneering in broiler research.

Therefore, this study aimed to evaluate the effects of phytase in broiler diets on zootechnical performance and diet cost through a technical economic analysis associated with a systematic review and meta-analysis.

## MATERIALS AND METHODS

### Selection of articles and eligibility criteria

**Figure 1:**
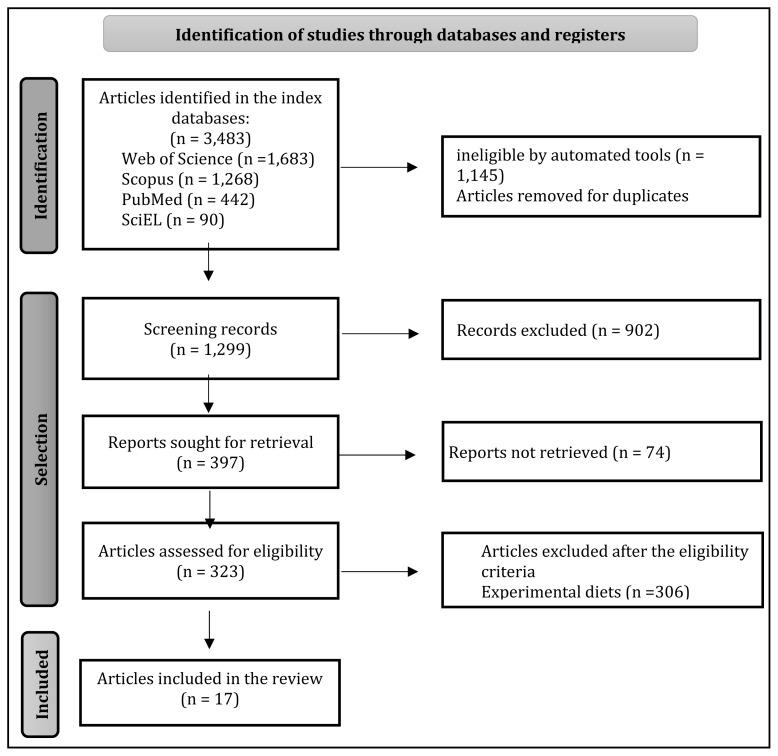
PRISMA flowchart. Adapted from Page et al [18] with CC-BY.

A database was created on the basis of the selection and collection of scientific articles indexed and published between 2018 and 2023. The research question was framed using the “PICo” strategy, identifying the “Population” as “broiler”, the “Interest” as “phytase”, and the “Context” as “performance” OR “42 days” [17]. The searches were performed on the Web of Science, Scopus, PubMed and SciEL platforms via the keywords “phytase” AND “broiler”. After the preliminary screening, the search criteria were further refined by incorporating the terms AND “Performance” AND “42 days” to narrow down relevant studies. The literature search was conducted from December 2022 to June 2023. All the articles found in each database were exported to EndNote Web, which made it possible to organize the bibliographic references and the removal of duplicate records obtained from the indexing databases. References from the identified publications were also reviewed to identify any additional relevant articles. The PRISMA (Preferred Reporting Items for Systematic Reviews and Meta-Analyses) flowchart was used to select the articles (Figure 1) [18].

For the choice of articles, the primary criterion defined was that only articles related to the performance of broilers supplemented with phytase would be considered eligible. The defined secondary eligibility criteria were as follows: a) *in vivo* studies; b) studies composed of at least three diets (a diet formulated according to nutritional requirements and free of phytase [BD], a diet formulated with a reduced level of digestible P of the nutritional matrix and without the inclusion of phytase [DRP] and a diet formulated with a reduction in digestible P from the nutritional matrix supplemented with phytase [DRP+PHY]); c) studies of performance in the initial, growth and final stages; and d) studies with a minimum experimental period of 42 days (birds between 1 and 42 days).

### Data systematization and experimental diets

The methodology applied for database construction and data coding followed the procedures described by Bougouin et al [19]. A total of 3.483 publications were identified, among which 17 publications were selected (Table 1) after applying the eligibility criteria. The data from the selected articles were tabulated and organized in electronic spreadsheets, and subsequently, coding was applied to the data from the initial, growth and final stages to facilitate explanation and interpretation. The same coding procedure was applied to group the experimental diets.

The number of participants was recorded (number of replicates per treatment and birds per replicate), as well as the phytase level (FTU/kg), the reduction in digestible P, the initial and final body weights of the birds, and the experimental period. In addition, the means and corresponding standard deviations for each performance variable were collected for the BD, DRP, and DRP+PHY treatments. These data were subsequently used to calculate the standardized mean differences (SMD), assess heterogeneity, and evaluate publication bias for each dependent variable.

For the technical-economic analysis, diets based on corn and soybean meal were formulated according to the nutritional requirements of male broilers with regular-medium performance (1–7; 8–21; 22–33; 34–42 days), according to the nutritional recommendations of Rostagno et al [37].

### Quotation of feed ingredients

Nutritional costs were generated from the feed formulations for each of the phases, and their effects on the performance results obtained in each study were evaluated. For this purpose, the ingredients were listed in the database of the Center for Advanced Studies in Applied Economics (CEPEA/Esalq/USP) and in the Chicago Stock Exchange through the average price of the ingredients (kg) from 2018–2023. Dicalcium phosphate, limestone, premix, aggregate, amino acids and phytase were quoted through different national suppliers, and their respective average prices were adopted.

The values quoted in reais were converted into dollars (US$ 1.00 = R$ 5.12) according to the average value obtained from the five-year history of the central bank: Corn = US$ 0.188, soybean meal = US$ 0.415, soybean oil = US$ 0.674, dicalcium phosphate = US$ 0.776, calcitic limestone = US$ 0.023, L-lysine = US$ 3.233, DL-methionine = US$ 7.759, L-threonine = US$ 5.990, L-tryptophan = US$ 16.784, Salt = US$ 0.508, Premix = US$ 10.906, inert = US$ 0.041, and phytase = US$ 17.142. The price of live chickens was US$ 0.922. Additionally, the model incorporated a PHY dose of 500 FTU/kg and the live broiler price provided by the local poultry agroindustry. FTU refers to phytase units and is defined as the amount of enzyme required to hydrolyze 1 μmol inorganic P per minute from 0.0051 mol/L sodium phytate at pH 5.5 and a temperature of 37°C [38].

### Growth performance of broiler

For performance, initial weight (IW), total weight gain (TWG), total feed intake (TFI), feed conversion ratio (FCR) and final weight (FW) were evaluated. The calculation for digestible P consumption (PC) was as follows: DB, PC, g = ([TFI×0.356]/100); DRP and DRP+PHY, PC, g = ([TFI× 0.206]/100). The following feed costs were analyzed: revenue, feed cost/broiler head, profit, gross margin (MB), feed cost/kg broiler, feed cost/ton of broiler and variation in feed costs. The variation in food cost was determined from the DRP and DRP+PHY diets in relation to the basal diet.

### Feed cost and economic indicators

Performance data from animals fed DB, DRP, and DRP+PHY diets were used in the equations, and the differences among the results were used to estimate the economic impact. Production costs (US$/kg) were established for each production phase, corresponding to the DB, DRP and DRP+PHY, respectively. The adopted values were: 1–7 days (0.369, 0.360, 0.373), 8–21 days (0.361, 0.352, 0.354), 22–33 days (0.351, 0.343, 0.344), and 34–42 days (0.328, 0.321, 0.322). The costs of the diets in US$ per phase were obtained from the following equations: Diet cost per phase (FDC) = cost/kg of the diet in the phase×TFI of the phase; Total diet cost (DTC): sum of FDC at all stages; Feed cost per broiler produced (Cost/head) = DTC/TWG; Feed cost per kg of chicken (Cost, U$/kg) = Cost/head/FW; Feed cost per ton of chicken = Cost, U$/kg× 1,000; Revenue = Price per kg of chicken (US$)×FW; Profit = revenue−Cost/kg; Gross margin = profit/revenue; Variation in feed cost DRP = ([Cost, U$/kg of DRP×100]/Cost, U$/kg of DB)−100; Variation in feed cost DRP+PHY = ([Cost, U$/kg of DRP+PHY×100]/Cost, U$/kg of DB)−100.

### Statistical analysis

The performance and economic data were analyzed using SAS software [39]. Analysis of means was performed using Fisher’s F-test, and when significant, means were compared via Tukey’s test at a 5% probability. Subsequently, twelve meta-analyses were conducted to assess the effect size among the BD vs. DRP, BD vs. DRP+PHY, and DRP vs. DRP+PHY groups for the dependent variables (TFI, TWG, FCR, and FW), using the SMD with a 95% confidence interval (CI). Calculations were performed using the escalc function in the METAFOR package [40], and mean effects were considered significant when the CIs did not include zero.

Heterogeneity and variation among studies were assessed using the significance levels of the chi-square test and the corresponding I^2^ statistic. Using the Borenstein et al [41] benchmark range for I^2^ statistics, I^2^<25%, 25%≤I^2^≤50%, 50%≤ I^2^<75%, and 75%≤I^2^≤100% were interpreted as representing low, moderate, high, and very high levels of heterogeneity, respectively. As described by Vieira et al [42], heterogeneity was considered significant at p<0.10, since the chi-square test has relatively low power to detect heterogeneity when the number of studies is small.

Regardless of the chi-square and I^2^ results, heterogeneity was accounted for in the meta-analysis by applying random effects models (study, broiler strain, diets, and P reduction) to estimate the overall effects of the groups on the selected variables and their statistical significance [42]. Publication bias in the meta-analysis was assessed using Egger’s test and was considered significant at p<0.05 [43]. SMD, heterogeneity and publication bias analyses were performed using the metafor-package (ver. 4.8-0) in R (ver. 4.5.1; R Foundation for Statistical Computing).

## RESULTS

### Database characterization

The seventeen articles included in the meta-analysis comprised a total population of 16.151 broiler chickens. The most common breed reported in the studies was Cobb 500 (n = 6), followed by Ross 308 (n = 4), Ross 708 (n = 2), Ross (n = 2), Cobb 400 (n = 1), Sosso (n = 1), and UK Chunky (n = 1). Most experimental diets were based on corn–soybean meal formulations (n = 11), while others used CSW (n = 1), CSF (n = 1), CSWG (n = 1), CSD (n = 1), and WSDR (n = 1) as basal diets. The average phytase supplementation was 1.664 FTU/kg. The average reduction in digestible P was 0.15%. The mean initial and FWs of the birds were 44.02±2.03 g and 2.731±0.57 g, respectively. The mean experimental period was 43.86 days.

### Growth performance of broiler

Compared with those fed DB or DRP+PHY, broilers fed DRP presented a reduction (p<0.05) in TFI, TWG, FW and worsening of FCR (Table 2). BD and DRP+PHY did not differ in these variables. Compared with those fed the DRP or DRP+ PHY diet, the broilers fed DB had a greater percentage (p< 0.05) of daily PC.

### Meta-analysis results

The effects of supplemental phytase on the growth performance of meat-type chickens from 1 to 42 days of age were summarized using random effects meta-analysis models. The SMD estimates for TFI, TWG, FCR, and FW in the BD vs. DRP, BD vs. DRP+PHY, and DRP vs. DRP+PHY comparisons, along with the corresponding heterogeneity estimates, are presented in Table 3.

Supplementation with DRP+PHY significantly increased TFI (SMD = 4.471, p<0.001), TWG (SMD = 2.720, p<0.001), and FW (SMD = 2.774, p<0.001), while also improving FCR (SMD = 1.718, p = 0.002) compared with the DRP group. Furthermore, it is evident that the DRP group impaired broiler performance, as meta-analysis comparing both groups (BD vs. DRP; DRP vs. DRP+PHY) confirmed an effect (p_SMD_< 0.001) for all variables studied. Overall, the results indicate that phytase supplementation improves broiler performance, whereas P reduced diets without phytase impair it.

The responses of TFI, TWG, and FW measured in our meta-analysis exhibited significant heterogeneity among studies (p<0.001, I^2^ = 96%), suggesting that the overall results for these variables may be influenced by one or more qualitative random factors used to categorize the dataset. In contrast, no significant heterogeneity was observed for FCR (p = 1.000, I^2^ = 0%), indicating consistency across studies, broiler strains, diets, and P reduction levels for this variable. With reference to the Borenstein et al [41] benchmark range for heterogeneity, the I^2^ statistics detected among analyzed outcomes in our meta-analysis were interpreted as representing very high levels of heterogeneity, as the proportions fell within 75%≤I^2^≤ 100%. To address this concern, it was imperative to explore what causes this heterogeneity.

The decomposition of heterogeneity for TWG among the groups indicated that broiler strain was the main source of variation in TWG (I^2^ = 54.3% to 65.6%), TFI (I^2^ = 57.04% to 92.52%), and FW (I^2^ = 60.34% to 68.25%). Diet contributed moderately to TWG (I^2^ = 13.9% to 27.9%), TFI (I^2^ = 0% to 38.05%), and FW (I^2^ = 10.77% to 21.47%), whereas the study had a smaller influence (I^2^ = 14.1% to 16.5%, 0% to 3.86%, and 14.47% to 16.99%, respectively). P reduction had minimal effect on variability (I^2^ = 0% to 1.42%). It is evident that the broiler strain was the main source of variation, whereas P reduction had a minimal effect on the variability of the results. No publication bias was detected across the variables.

### Feed cost and economic indicators of broilers

Broilers fed DRP had lower (p<0.05) revenue, cost/head and profit than those that consumed DB or DRP+PHY did (Table 4). The birds fed DRP+PHY had lower (p<0.05) costs/heads than did those fed DB, but there was no difference in revenue or profit. There were no effects of the diets on BM, cost/kg, cost/ton or variation.

## DISCUSSION

The effects of exogenous phytase supplementation on broiler performance have been well documented [4,15,25]. Previous meta-analyses have examined the impact of phytase supplementation on broiler performance [7,19,42,44]. However, to date, no other meta-analysis has specifically reported the effects of phytase supplementation on broiler performance and economic impacts, using a systematic review and meta-analytic approach combined with economic modeling at both farm and global scales.

The results of the present study demonstrate that broilers fed diets with reduced P levels supplemented with phytase exhibit similar performance to that of animals fed diets formulated with a nutritional level of P and without phytase. However, it was found that manipulated diets with reduced nutritional P levels and not supplemented with phytase impair the performance of broilers.

The heterogeneity observed for TFI, TWG, and FW (I^2^>96%) highlights the influence of broiler strain, diets, and P reduction on these outcomes, whereas FCR exhibited remarkable consistency across studies (I^2^ = 0%). No significant publication bias was detected, reinforcing the reliability and validity of the evidence from our meta-analysis in estimating the overall effect size of phytase supplementation in reduced P diets for broilers.

In general, phytase supplementation hydrolyzes phytate, mitigating its antinutritional effects and enhancing P availability and digestibility. As a result, phytase consistently improves growth performance and nutrient utilization in broilers fed P-deficient diets [15].

Thus, we can infer that phytase supplementation efficiently provided the phytic P contained in the plant ingredients of the diets, since the TFI, TWG, FCR and FW of the broilers fed DRP+PHY were greater than those of the animals fed DRP. The diets formulated with P reduction and without phytase reduced the TFI and TWG and consequently impaired the FCR and FW of the broilers. P intake was lower for birds fed DRP and DRP+PHY than for those fed DB.

The results of the present study are in agreement with those reported by Freitas et al [44], who evaluated increasing levels of phytase (500, 1,000 and 1,500 FTU/kg) in the diets of broilers with reduced P (0.150%), Ca (0.165%) and Na (0.035%) in the nutritional matrix and did not observe any difference in the performance of the animals compared with those that consumed DB.

Importantly, compared with DB, DRP+PHY improved the FCR, even at a lower concentration and intake of P, which shows that phytase supplementation potentiated the use of nutrients from the diet by the animals, which were used for their full development, as reflected in heavier broilers than DRPs without phytase. The better use of nutrients and reduced intake of inorganic P also suggest lower excretion of P, N and other polluting compounds into the environment, indicating the contribution of phytase to reducing the polluting effects of poultry production, as observed by Moradi et al [5].

The phytate present in ingredients of plant origin is considered an antinutritional factor that restricts the absorption and digestion of minerals and nutrients, worsening the performance of birds. In addition, inadequate P intake by an animal may impair its performance. According to McDowell [46], loss of appetite is one of the first clinical signs observed in birds with P deficiency, which reduces feed intake. P also participates fully in energy metabolism and ATP production, and its nutritional deficiency can promote decreased weight gain, worsening FCR and, consequently, a decrease in FW in birds, which may explain the negative effects caused by RPD observed in the present study.

On the other hand, the positive performance results observed through phytase supplementation in the diet with reduced P for broilers are probably related to the increased availability of P [29]. Phytase is able to hydrolyze the phytic acid molecules contained in ingredients of plant origin, releasing P and other minerals, such as calcium, sodium, zinc, iron and magnesium, as well as amino acids, such as glycine, serine, threonine and proline, in complex with the phytase molecule phytate, making them available for absorption and metabolism [47].

The DRP+PHY diet did not negatively affect performance, so it was similar to DB, suggesting that it is possible to perform partial or total replacement of inorganic P sources in the initial, growth and final phases in such a way that this attractive nutritional strategy enables production, reducing the nutritional cost. Supplementing broiler diets with 500 FTU/kg of PHY can reduce production costs by 0.81% per kilogram of live weight compared to diets without PHY [12].

The economic modeling in our study showed that the economic indicators of revenue and profit were reduced when the animals were fed DRP. The DB and DRP+PHY generated similar revenues and profits. When we analyzed the cost/head, we observed that the inclusion of phytase was reduced (p<0.05) by US$ −0.057 compared with that in the animals that received DB. The animals fed DRP+F produced more profit per head than did those fed DB or DRP, with values of US$ 0.015 and US$ 0.075, respectively.

Importantly, although phytase did not affect the nutritional cost/kg or the nutritional cost/ton in the present study, when we analyzed the mean values of cost/ton, we observed that, compared with DB and DRP, DRP+PHY was able to reduce the cost/ton by US$ −9.523 and −10.484, respectively, without worsening the performance of the animals.

Broiler chickens do not produce the enzyme phytase, which is responsible for hydrolyzing and digesting phytate. Therefore, most of the P present in phytate is excreted without being absorbed. In agricultural feed ingredients such as wheat, corn, and soybean meal, approximately 75% of the total P is present in the form of phytate compounds, which, in the absence of phytase, are excreted [15]. The studies included in our meta-analysis used diets based on ingredients with considerable phytate content, suggesting that the P that would otherwise be excreted was made available to the birds through phytase supplementation, even in diets with reduced P levels, thereby contributing to lower nutritional costs.

Considering the perspective of the industrial scale of world broiler production, the inclusion of phytase with a reduction in digestible P in the nutritional matrix of the diet can clearly impact the cost of the feed because it decreases the cost of the diet per ton of broiler produced in US$ 9.523. According to the USDA forecast, in 2023, the world production of chicken meat increased by 1.8%, approaching 103 million tons produced, which represents a reduction of 980 million dollars. In turn, in Brazil, considering the production of 14.745 million tons produced in 2023, this would represent a decrease in the nutritional cost of approximately US$ 140.4 thousand.

In addition, the mean values of BM and cost variation changed among the treatments, in which DRP+PHY promoted higher BMs than BD and DRP did and resulted in negative cost variation, which indicates good financial performance and operational efficiency of the diet, enabling its use in broilers.

These economic performance indicators are important for guiding producers in decision making on which administrative and nutritional plans to adopt, ensuring the most rational use of financial resources and production factors to obtain better economic results [48]. In the present study, satisfactory results were clearly observed, which suggests the inclusion of phytase in the diet of broilers.

Considering the global impacts of phytase on diet costs, Moss et al [49], when using phytase (1,000 FTU/kg) in broiler chicken feed for 42 days, reported that the use of the enzyme together with its nutritional matrix reduced the cost of the diet by US$ 44.20 per ton. Considering the total volume of feed produced worldwide of 351 million tons for broilers, this would be equivalent to a reduction of 15.5 billion dollars in nutritional costs. These weights indicate the magnitude of the economic impacts of the inclusion of phytase and its potential to contribute to the global production of chicken meat.

The cost results of the inclusion of phytase in diets for broilers corroborate the findings of [11,41], in which both reported a reduction in costs when the diets were formulated with phytase and were nutritionally reduced in Ca, P, metabolizable energy and crude protein. Nascimento et al [11] proposed that, as phytase levels of 1,000 and 1,500 FTU/kg and their respective suggested nutritional reductions in the nutritional matrix of the enzyme are included, it is possible to decrease costs by US$ 3.58 and US$ 6.12 per ton.

Nutritional strategies that seek to reduce the cost of feed and improve the profitability of poultry production, among which the nutritional adjustments associated with enzymatic inclusion are efficient, have been widely used. However, drastic nutritional reductions can decrease nutrient availability and negatively impact animal performance. Thus, the results of this study show that reducing the digestible P level of the nutritional matrix of a diet supplemented with phytase can maintain the performance standard of broilers and reduce the nutritional cost of the diet.

Notably, the nutritional reduction and the ingredients used in the diets formulated for this study contributed to the positive results of the nutritional cost. In industrial poultry, reductions in costs and increases in revenue, even if small as those obtained in the present study, are extremely relevant, since this production chain generates high movements and the capital generated is very high. Thus, we can infer that the possibility of a 0.15% reduction in digestible P from the nutritional matrix and the reduction in P from inorganic sources in the diet can promote a reduction in feed cost without negatively affecting the performance of broilers.

## CONCLUSION

Our study confirms that reducing dietary P without phytase supplementation impairs broiler performance. However, when phytase supplementation is adopted in reduced P diets, performance is maintained and nutritional costs are lowered, resulting in a saving of approximately US$ 9.52 per ton of broilers produced. It should be emphasized that the economic benefits are achieved when phytase is included in diets formulated with nutrition reductions (P reduced), nevertheless, such reductions must be applied cautiously, as abrupt decreases compromise broiler performance. A 0.15% reduction in digestible P in the nutritional matrix of the diet and phytase supplementation are suggested. These findings provide practical valuable information for the poultry industry to optimize feed formulations, significantly reducing feeding costs without compromising performance and in a sustainable way.

## Figures and Tables

**Table 1:** Characteristics of the studies used in the technical economic study on the use of phytase in diets for broilers (1–42 d of age)

Studys	Animals No.	Broiler strain	Diets	P reduction (%)	Phytase levels (FTU/kg)	Origin of phytase
Broch et al [20]	920	Cobb 500	CSW	0.14	0–1,000/3,000	*Aspergillus oryzae*
Dimas et al [21]	792	Cobb 500	Corn-soybean	0.15	0–4,500	*Aspergillus oryzae*
Dessimoni et al [22]	672	Cobb 500	Corn-soybean	0.14	0–500	*Escherichia coli*
Lee et al [23]	2,970	Cobb 400	Corn-soybean	0.15	0–500/3,000	*Escherichia coli*
Ribeiro et al [24]	792	Ross	Corn-soybean	0.15	0–1,000/4,000	*Aspergillus oryzae*
Attia et al [25]	360	Sasso	CSF	0.15	0–500	*Aspergillus niger*
Babatunde et al [26]	2,400	Ross 308	Corn-soybean	0.15	0–250/1,000	*Aspergillus niger*
Taheri et al [27]	440	Ross 308	Corn-soybean	0.25	0–1,500/3,000	*Escherichia coli*
Broch et al [28]	875	Cobb 500	CSWG	0.15	0–500/1,500	-
Kriseldi et al [29]	1,024	Ross 708	Corn-soybean	0.15	0–4,500	*Escherichia coli*
Bertechini et al [30]	1,000	Cobb 500	Corn-soybean	0.20	0–500	-
Gulizia et al [31]	1,200	Ross 708	CSD	0.20	0–500/2,000	*Escherichia coli*
Javadi et al [32]	490	Ross	CSW	0.10	0–250/1,000	*Serratia odorifera*
Mulvenna et al [33]	308	Ross 308	WSDR	0.22	0–500/1,500	*Escherichia coli*
Shi et al [34]	528	Ross 308	Corn-soybean	0.10	0–500/10,000	*Trichoderma strain*
Shimeno et al [35]	900	UK chunky	Corn-soybean	0.10	0–500	*Aspergillus oryzae*
Maynard et al [36]	480	Cobb 500	Corn-soybean	0.15	0–2,000	*Escherichia coli*

CSW, corn-soybean-wheat; CSF, corn-soybean-fish meal; CSWG, corn-soybean-wheat-gluten; CSD, corn-soybean; WSDR, wheat-soybean-DDGS-rapeseed.

**Table 2:** Performance of broilers (1–42 d) fed diets with or without phytase

Variables	Diets			SEM	p-value

	DB	DRP	DRP+PHY		
IW (g)	43.955	43.932	44.191	0.106	0.887
TFI (kg)	4.518^a^	4.242^b^	4.460^a^	0.102	0.001
PC (%)	0.356^a^	0.206^b^	0.206^b^	0.000	0.001
PC (kg)	0.016^a^	0.009^b^	0.009^b^	0.000	0.001
TWG (kg)	2.770^a^	2.550^b^	2.721^a^	0.079	0.001
FCR (kg/kg)	1.661^b^	1.703^a^	1.665^b^	0.030	0.016
FW (kg)	2.816^a^	2.603^b^	2.773^a^	0.080	0.001

a,b Means followed by different letters in the columns differ (p<0.05) according to Tukey’s test.

DB, basal diet; DRP, reduced P diet, DRP+PHY, DRP diet supplemented with phytase; SEM, standard error of means; IW, initial weight; TFI, total feed intake; PC, P consumption; TWG, total weight gain; FCR, feed conversion ratio; FW, final weight.

**Table 3:** Summary of standardized mean difference for the performance variables included in the meta-analysis and their analyses by different groups (diets)

Diets	N° of studys (k)	Random effects model			Heterogeneity	

		SMD	95% CI	p-value	I^2^ (%)	p-value
Total feed intake (kg)						
DB×DRP	17	4.471	3.69–5.25	<0.001	96	<0.001
DB×DRP+PHY	17	4.471	3.69–5.25	<0.001	96	<0.001
DRP×DRP+PHY	17	4.471	3.69–5.25	<0.001	96	<0.001
Total weight gain (kg)						
DB×DRP	17	2.719	2.07–3.36	<0.001	96	<0.001
DB×DRP+PHY	17	2.72	2.07–3.37	<0.001	96	<0.001
DRP×DRP+PHY	17	2.72	2.07–3.38	<0.001	96	<0.001
Feed conversion ratio (Kg/Kg)						
DB×DRP	17	1.718	0.65–2.78	0.002	0	1.000
DB×DRP+PHY	17	1.718	0.65–2.78	0.002	0	1.000
DRP×DRP+PHY	17	1.718	0.65–2.78	0.002	0	1.000
Final weight (kg)						
DB×DRP	17	2.774	2.13–3.41	<0.001	96	<0.001
DB×DRP+PHY	17	2.774	2.13–3.41	<0.001	96	<0.001
DRP×DRP+PHY	17	2.774	2.13–3.41	<0.001	96	<0.001

SMD, standardized mean difference; CI, confidence interval; DB, basal diet; DRP, reduced P diet; DRP+PHY, DRP diet supplemented with phytase.

**Table 4:** Feed cost and economic indicators of broilers (1–42 d old) supplemented with or without phytase

Variables	Diets			SEM	p-value

	DB	DRP	DRP+PHY		
Revenue (U$)	2.598^a^	2.400^b^	2.557^a^	0.073	0.001
Cost/head (U$)	1.662^a^	1.523^c^	1.605^b^	0.037	0.001
Profit (U$)	0.936^a^	0.876^b^	0.951^a^	0.045	0.001
MB (%)	35.118	35.186	36.200	0.011	0.120
Cost (U$/kg)	0.609	0.610	0.599	0.010	0.114
Cost (U$/ton)	609.220	610.181	599.697	10.891	0.114
Variation (%)	0.000	0.164	−1.642	0.040	0.140

a–c Means followed by different letters in the columns differ (p<0.05) according to Tukey’s test.

DB, basal diet; DRP, reduced P diet; DRP+PHY, DRP diet supplemented with phytase; SEM, standard error of means; MB, gross margin.

## Data Availability

Upon reasonable request, the datasets of this study can be available from the corresponding author.
